# Direct dating reveals the early history of opium poppy in western Europe

**DOI:** 10.1038/s41598-020-76924-3

**Published:** 2020-11-20

**Authors:** Aurélie Salavert, Antoine Zazzo, Lucie Martin, Ferran Antolín, Caroline Gauthier, François Thil, Olivier Tombret, Laurent Bouby, Claire Manen, Mario Mineo, Aldona Mueller-Bieniek, Raquel Piqué, Mauro Rottoli, Núria Rovira, Françoise Toulemonde, Ivana Vostrovská

**Affiliations:** 1 Unité Mixte de Recherche (UMR) Archéozoologie, Archéobotanique: Sociétés, Pratiques et Environnements, Muséum National d’Histoire Naturelle (MNHN), Centre National de Recherche Scientifique (CNRS), Alliance Sorbonne Université, Paris, France; 2 Laboratory of Prehistoric Archaeology and Anthropology, University of Geneva, Geneva, Switzerland; 3 UMR EDYTEM, CNRS, Université Grenoble Alpes, Université Savoie Mont-Blanc, Chambéry, France; 4 Integrative Prehistory and Archaeological Science (IPAS), Universität Basel, Basel, Switzerland; 5 Laboratoire des Sciences du Climat et de l’Environnement (LSCE), CNRS, Commissariat à l’Energie Atomique (CEA), Université de Versailles Saint-Quentin, Paris-Saclay, Paris, France; 6 UMR Histoire Naturelle de L’Homme Préhistorique, Musée de L’Homme, MNHN, Paris, France; 7 ISEM, Univ Montpellier, CNRS, EPHE, IRD, Montpellier, France; 8 UMR TRACES, CNRS, Université Toulouse Jean Jaurès, Toulouse, France; 9 Museo delle Civiltà - Museo Preistorico Etnografico “Luigi Pigorini”, Rome, Italy; 10 W. Szafer Institute of Botany, Polish Academy of Sciences, Kraków, Poland; 11 Departament de Prehistòria, Universitat Autònoma de Barcelona, Bellaterra, Spain; 12 Laboratorio Di Archeobiologia Dei Musei Civici Di Como, Department of Humanities, Università Degli Studi Di Trento, Trento, Italy; 13 UMR Archéologie Des Sociétés Méditerranéennes, Université Paul-Valéry Montpellier 3, CNRS, Ministère de La Culture Et de La Communication, Inrap/LabEx Archimède, Montpellier, France; 14 Faculty of Arts, Palacký University Olomouc, Olomouc, Czech Republic

**Keywords:** Plant domestication, Biogeochemistry

## Abstract

This paper aims to define the first chrono-cultural framework on the domestication and early diffusion of the opium poppy using small-sized botanical remains from archaeological sites, opening the way to directly date minute short-lived botanical samples. We produced the initial set of radiocarbon dates directly from the opium poppy remains of eleven Neolithic sites (5900–3500 cal BCE) in the central and western Mediterranean, northwestern temperate Europe, and the western Alps. When possible, we also dated the macrobotanical remains originating from the same sediment sample. In total, 22 samples were taken into account, including 12 dates directly obtained from opium poppy remains. The radiocarbon chronology ranges from 5622 to 4050 cal BCE. The results show that opium poppy is present from at least the middle of the sixth millennium in the Mediterranean, where it possibly grew naturally and was cultivated by pioneer Neolithic communities. Its dispersal outside of its native area was early, being found west of the Rhine in 5300–5200 cal BCE. It was introduced to the western Alps around 5000–4800 cal BCE, becoming widespread from the second half of the fifth millennium. This research evidences different rhythms in the introduction of opium poppy in western Europe.

## Introduction

Nowadays, the opium poppy has the ability to grow in most parts of the world, regardless of soil properties, temperature, or topography. Moreover, the cultivated opium poppy (i.e. *Papaver somniferum* subsp. *somniferum* L.) is largely grown for medicinal, psychoactive, and alimentary uses^1^. Despite its importance for human societies, the history of the plant has not been the subject of detailed studies. This paper presents the results of a project that aims to define the chrono-cultural framework of the domestication and early diffusion of the species using botanical remains from archaeological sites (i.e. the seeds and sometimes the stigmatic discs, charred or waterlogged) and radiocarbon dating techniques^2^.

The kidney-shaped seed of the opium poppy measures less than 1 mm in diameter. Thanks to optimized sampling and sieving methods, seeds are now regularly identified in archaeobotanical samples from western Europe. Spontaneous in the central and western Mediterranean Basin, *P. somniferum* subsp. *setigerum* (DC.) Arcang. is assumed to be the wild relative of the cultivated *P. somniferum* subsp. *somniferum* L., even if the issues concerning its taxonomy and phylogeny have not been entirely resolved^3–5^. The modern and archaeological seeds of both *P. somniferum* subspecies cannot yet be differentiated based on their size, morphology, or outer integument ornamentation^6^. For this reason, it is impossible to tell whether archaeological specimens correspond to wild poppies, or cultivated varieties.

**Figure 1:**
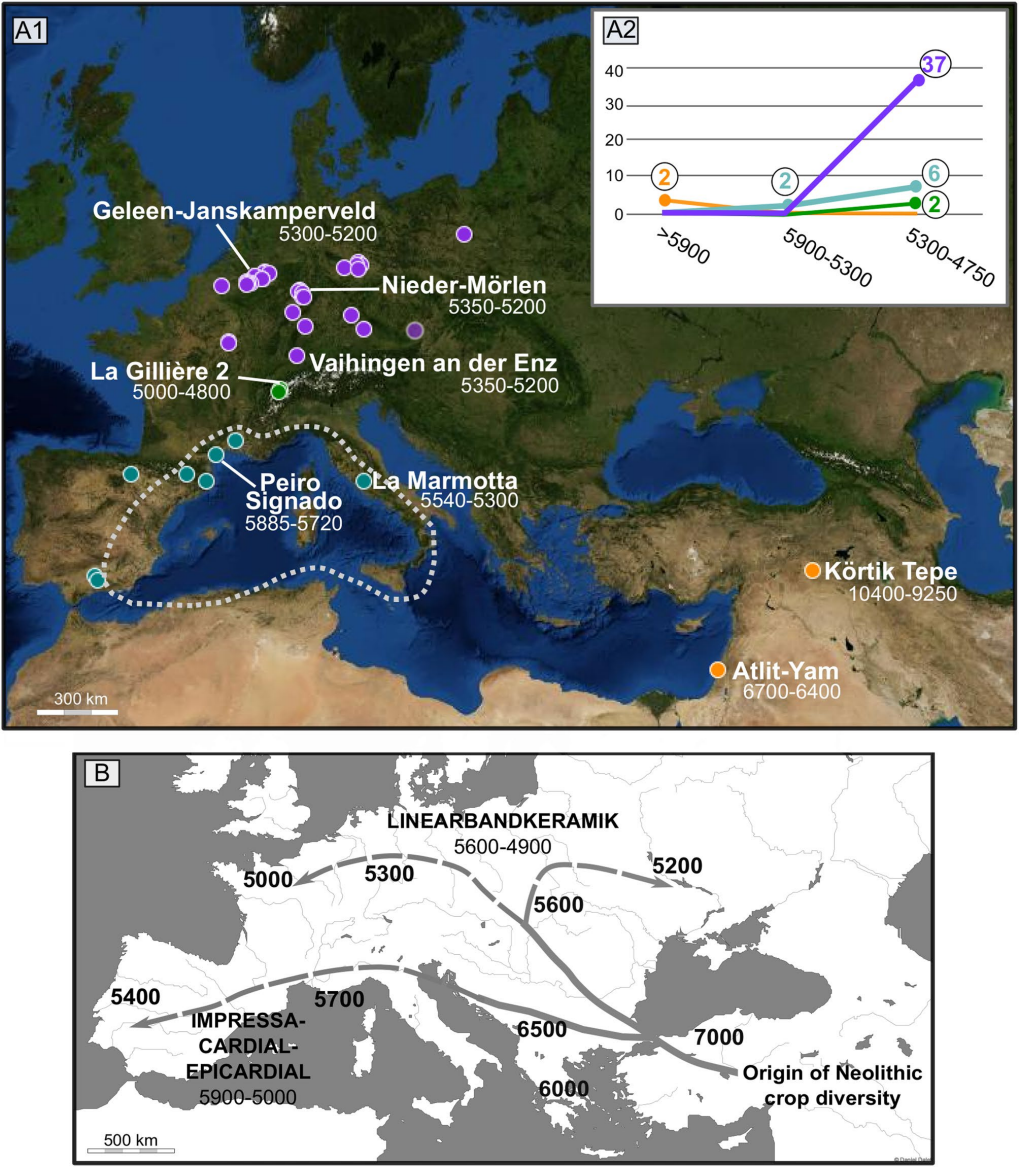
(A1) Dataset of the Early Neolithic sites where opium poppy remains have been identified (Supplementary Information S1) with the locations of the earliest records in the Near East (orange circles), Mediterranean (blue circles), temperate Europe (purple circles), the western Alps (green circles), and current wild poppy populations (dotted line)^11^. The open access map was created with umap, an OpenStreetMap project (version 1.2.2), under ODbL 1.0 license. Contains credits: A. Salavert (AASPE, MNHN-CNRS), Map background credits: NASA 2016: https://umap.openstreetmap.fr/fr/map/salavertetal_sr_fig12_460185. (A2) Number of Early Neolithic sites where the plant is identified by chronological ranges corresponding to the period before the arrival of the first European farmers (< 5900 BCE); to the beginning of the Early Neolithic in the Mediterranean and temperate Europe (5900–5300 BCE); to the second stage of the LBK (LBK II–V) and the beginning of the Early Neolithic in the western Alps until the end of the period in western Europe (5300–4750 BCE).; (B) Overview of the spatial and temporal framework for the diffusion of the “Neolithic crop package” from the Near East to western Europe and the two European pioneer Neolithic complexes. The main chronological points are in cal BCE^18^. Contains credits: A. Salavert (AASPE, MNHN-CNRS), Map background credits: D. Dalet.

In the last ten years, several scenarios have been proposed to assess the origin of the crop, relying on the inventories of early archaeological attestations of the plant in western Europe and the Near East, as well as the geographical distribution of *P. somniferum* subsp. *setigerum*^7–11^. The chronological framework has mainly been supported by radiocarbon dating using both short-lived (animal bones, annual plants) and long-lived (charcoal, wood) biofacts, but ignoring the opium poppy remains themselves. To date, the hypothesis of its Near Eastern origin and diffusion to western Europe, together with Neolithic founder crops (e.g. emmer, einkorn, barley), is not well supported by archaeobotanical evidence. Only two Pre-Pottery Neolithic sites have delivered opium poppy seeds in the Near East and Anatolia^9,12,13^ (Fig. 1A1). However, several lines of evidence suggest that these seeds may be intrusive and come from more recent cultural layers; first, wild opium poppy does not grow today in the Near East or Anatolia^14,15^; secondly, no additional evidence was found despite the large amount of archaeobotanical studies in the area^16,17^, and thirdly, archaeological remains are currently absent on the Neolithic economy dispersal route from the Near East to western Europe, i.e. in the Balkans and central Europe prior to 5300 cal BCE^9^ (Fig. 1A1, Fig. 1B). The best argued hypothesis is that the opium poppy could be the only crop to have been domesticated in western Europe, given that 50 Early Neolithic sites (5900–4700 cal BCE, Fig. 1A2, see Supplementary Information S1, all calibrated radiocarbon dates are given with a 2-sigma range) with at least one opium poppy seed have been recorded through archaeobotanical literature^10,11,18–21^. The two earliest attestations are located in the Mediterranean, where eight sites, dated between ca. 5900 and 5000 BCE, have delivered opium poppy remains (Fig. 1A1). In addition, the wild opium poppy is currently distributed throughout the central and western Mediterranean^11^. At Peiro Signado (Impressa culture), in southern France, a sole charred seed has been identified. This open-air site has been dated using charcoal and cereals to between 5885 and 5720 cal BCE^22,23^. At La Marmotta (Cardial culture), in central Italy, charred capsules were preserved in several archaeological layers^24^. Part of the multiphase pile-dwelling site is dated to between 5538 and 5290 cal BCE by dendrochronology^25^. Additional records in Spain and southeast France are mainly attributed to the Cardial and Epicardial cultures and dated from 5200 and 5000 BCE^7,22^. In temperate Europe (i.e. central and northwestern continental Europe), nearly 40 sites delivered opium poppy remains (Fig. 1A2, See Supplementary Information S1). These sites are mainly attributed to the second stage of the Linearbandkeramik (LBK) period (LBK II–V), around 5300–5000 cal BCE^10^. To date, there are no recorded remains from sites attributed to the earliest LBK period (LBK I), ca. 5600–5300 cal BCE in central Europe^18,26^. In this cultural complex, the earliest attestations are the charred seeds discovered in structures dated to the Flomborn phase from ca. 5300 cal BCE (LBK II, Fig. 1A1)^27–29^. The northwards diffusion (i.e. outside of its native ecological range) would have occurred through contacts with Neolithic Cardial populations from the Mediterranean^11,26^. In the western Alps, the data on the Neolithization process are still incomplete. The earliest attestations are located at La Gillière 2 and Tourbillon, both belonging to the so-called *Néolithique ancien valaisan*, and dated between ca. 5000 and 4850 cal BCE^20^. Here, the ceramics and botanical macroremains show some affinities with the sites of northern Italy^30–32^. To date, the opium poppy has not been identified on Mesolithic sites in either Mediterranean, temperate Europe or western Alps.

The scenario of a European origin for opium poppy is the most likely, but the chronological framework is based on indirect chrono-geographical landmarks either inferred from the archaeological context or the radiocarbon dating of associated material. As a result, possible intrusions cannot be discounted. Indeed, small seeds are likely to suffer from post-depositional movements and could therefore be intrusive, especially on sites which present successive settlement phases, with the few seeds recorded often originating from a minimal number of samples. Recent work on the chronology of the introduction of broomcorn millet (*Panicum miliaceum* L.) into Europe showed that the chronological attribution of many remains, based on their stratigraphic context, was incorrect^33^. Until very recently, the direct dating of opium poppy seeds was deemed impossible due to their size (typically less than 10–30 µg/seed). Today, the advent of a new generation of compact Accelerator Mass Spectrometry (AMS), together with optimized preparation protocols has allowed the minimum sample amount required to date archaeological events to be significantly reduced^34^. The dating of opium poppy seeds still represents a technical and methodological challenge, however, due to the diminutive size of these archaeobotanical remains.

**Figure 2:**
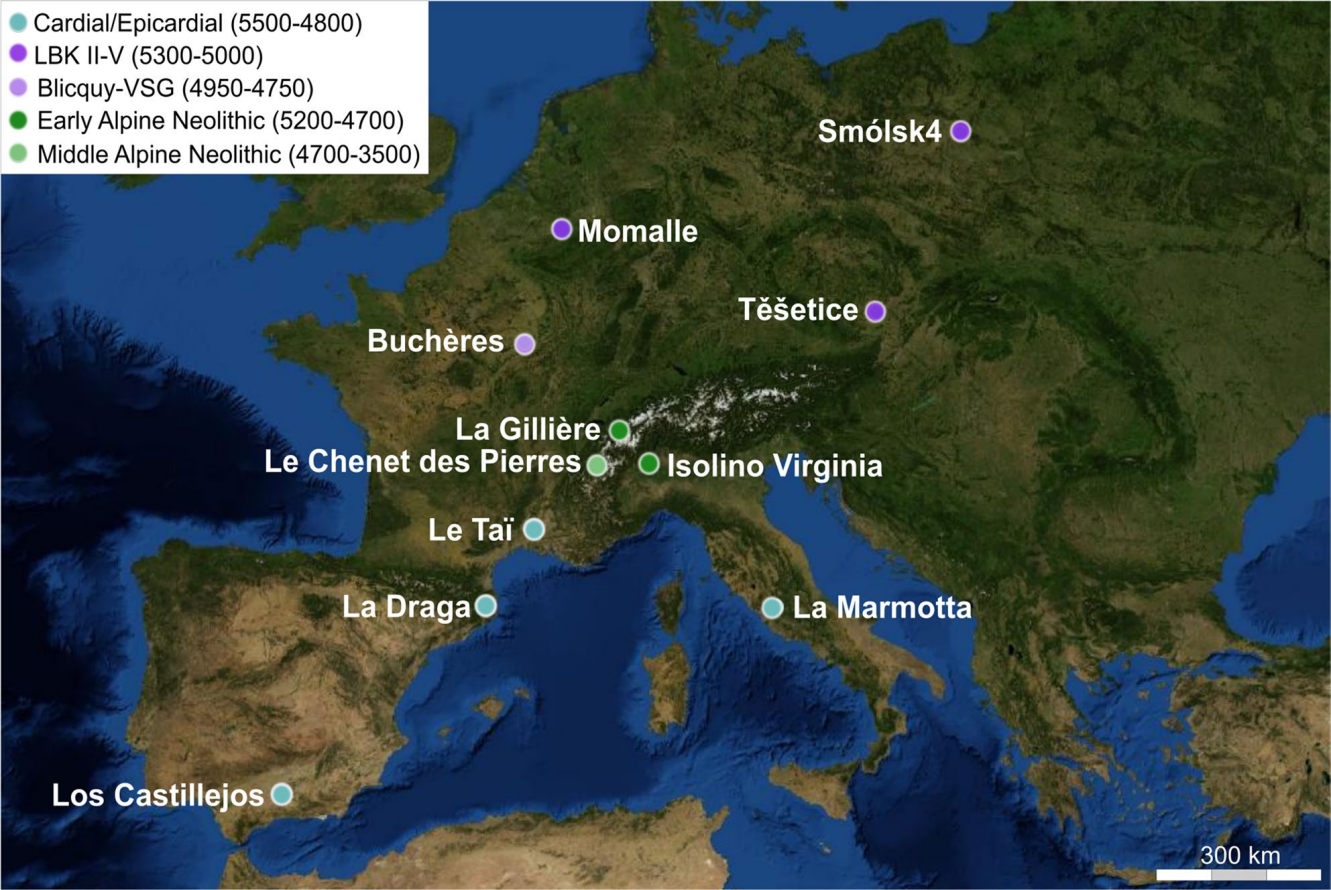
Location of the selected sites included in the dating program and chrono-cultural attributions based on radiocarbon dates indirectly performed on the opium poppy (absolute chronology) as well as artifacts (relative chronology). The chronological ranges are those of cultural facies. VSG: Villeneuve-St-Germain, LBK: Linearbandkeramik. The open access map was created with umap, an OpenStreetMap project (version 1.2.2), under ODbL 1.0 license. Contains credits: A. Salavert (AASPE, MNHN-CNRS), Map background credits: NASA 2016: https://umap.openstreetmap.fr/fr/map/salavertetal_sr_fig12_460185.

In this paper, we will test the above mentioned state of the art by directly dating (1) the antiquity of La Marmotta’s capsules (ca. 5500 cal BCE), to set an objective starting point for the use of opium poppy by pioneer farmers; (2) the early integration of the species in the LBK Neolithic economy (ca. 5300 cal BCE) from northwestern temperate Europe, and (3) the arrival of opium poppy in the western Alps between 5000 and 4850 cal BCE. For this purpose, eleven archaeological sites, attributed to the Neolithic period (5900–3500 cal BCE) were selected (see Supplementary Information S2). These sites potentially provide some of the earliest known opium poppy records in western Europe (Fig. 2).

## Material

### Site Selection

The sites were chosen based on the chronological attribution of the structure/layer where the opium poppy remains were recovered, its location, and the proficiency of the archaeological contextual information (Supplementary Information S2). Four sites (among the nine sites listed in the inventory, see Supplementary Information S2)—La Marmotta (Italy), La Draga (Spain), Le Taï (France), and Los Castillejos (Spain)—belong to the Impressed ware complex that corresponds to the first agro-pastoralists of the central and western Mediterranean. La Marmotta is one of the earliest sites that have delivered opium poppy in western Europe^22,24^ (Fig. 1A1). Three sites—Těšetice-Kyjovice (Czech Republic), Remicourt-Fond de Momalle (Belgium) and Smólsk 4 (Poland)—are spread over the geographical extent of the LBK culture (LBK II–V). These sites will make it possible to verify the presence of the opium poppy throughout the LBK territory, from Belgium to Poland, as well as its potential presence from the very beginning of the second LBK phase (ca. 5300 cal BCE) west of the Rhine. One site—Buchères-les Terriers (France)—is attributed to the Blicquy/Villeneuve-Saint-Germain culture (BVSG) that corresponds to the end of the Early Neolithic period (4950–4750 cal BCE) in northwest temperate Europe. This site will make it possible to control the presence of the opium poppy from the Early Neolithic in the north of France, where very few sites have delivered the species for this period. In the western Alps, La Gillière 2 (Switzerland) is one of the two early Neolithic sites (5200–5000 cal BCE) where the plant has been identified in this area (Fig. 1A1). On the southern Alpine foothills, Isolino Virginia (Italy) is the earliest Pre-Alpine pile-dwelling site, dating back to 5000 cal BCE^35^, to have delivered the species. Finally, Le Chenet des Pierres (France), in the northern French Alps, belongs to the Middle Neolithic period (4400–4200 cal BCE) and constitutes the most recent site of our dataset. These western alpine sites will contribute to the question of the late integration of the opium poppy in the Alpine Massif.

### Sampling

The samples were found in archaeological sediments from pits or ground levels, mixed with everyday life artifacts (i.e. potsherds, lithic implements, animal bones, crops, weeds). Due to the small size of the charred and/or waterlogged opium poppy seeds, referred to thereafter as “microsamples”, radiocarbon measurements are associated with a large error of ± 50 to ± 150 ^14^C years (yr), leading to wide uncertainty in calibrated ranges (i.e*.* 200–400 calendar yr with a 95.4% confidence level, Table 1). In order to reduce this uncertainty and to test for possible intrusion of microsamples, we also dated, when possible, a macrobotanical remain originating from the same sediment sample as the opium poppy, referred to thereafter as “macrosample” (Table 1). At Los Castillejos, the macrosample did not come from the same sediment sample as the microsample, but was deemed contemporaneous with the opium poppy remains as they both belong to the same stratigraphic horizon. Most of the macrosamples corresponded to annual plants (crops, weed). One sample from La Marmotta, composed of weighty opium poppy capsule fragments, was considered and processed as a macrosample. At Remicourt-Fond de Momalle, the macrosample corresponded to long-lived taxa (charcoal). The age of this sample may be affected by the old-wood effect if the charcoal came from the earliest tree rings, knowing that this tree species can have a lifespan of 150–200 years^36^. For Isolino Virginia, we have used a published date carried out on short-lived botanical remains^35^, and for La Draga one unpublished date generated within the AgriChange project^37^.

**Table 1:** Results of the radiocarbon dates performed on the micro- and macrosamples. The calibrated dates are presented with a 2-sigma error.

Site name	Country	Chronoculture	Processing	Type of sample	Taxon	Sample label	^14^C age (yr BP)	error (yr)	Cal BCE (95.4% confidence level)	Program/ Ref
Los Castillejos-Las Peñas de los Gitanos	Spain	Early Neolithic-Cardial	Microsample	Seeds	*Papaver somniferum*	ECHo2443	4330	70	3329–2704	Fyssen Program
			Macrosample	Seeds	*Hordeum vulgare* var. *nudum*	ECHo2260	6150	30	5209–5005	Fyssen Program
Remoulins-Le Taï	France	Early Neolithic-Epicardial	Microsample	Seeds	*Papaver somniferum*	ECHo2447	6140	100	5311–4804	Fyssen Program
			Macrosample	Seeds	*Triticum* sp.	ECHo2264	6150	30	5209–5005	Fyssen Program
La Draga	Spain	Early Neolithic-Late cardial	Microsample	Seeds	*Papaver somniferum*	ECHo2448	6090	90	5292–4791	Fyssen Program
			Microsample	Seeds	*Papaver somniferum*	ECHo2453	6060	110	5296–4717	Fyssen Program
			Macrosample	Seeds	*Triticum aestivum/durum/turgidum*	ETH-88875	6110	25	5207–4945	Unpublished, AgriChange project
La Marmotta	Italy	Early Neolithic-Cardial	Microsample	Capsules	*Papaver somniferum*	ECHo2454	6600	50	5622–5478	Fyssen Program
			Macrosample	Capsules	*Papaver somniferum*	ECHo2657	6600	30	5617–5480	Fyssen Program
Remicourt-Fond de Momalle	Belgium	Early Neolithic-LBK	Microsample	Seeds	*Papaver somniferum*	ECHo2446	6150	80	5305–4850	Fyssen Program
			Macrosample	Charcoal	*Fraxinus*	ECHo2263	6295	30	5329–5211	Fyssen Program
Buchères-les Terriers, Parc logistique de l'Aube 2013	France	Early Neolithic-BVSG	Microsample	Seeds	*Papaver somniferum*	ECHo2890	5840	60	4837–4546	Fyssen Program
			Macrosample	Seeds	*Pisum sativum*	ECHo2262	6000	30	4988–4797	Fyssen Program
Smólsk 4	Poland	Early Neolithic-LBK	Microsample	Seeds	*Papaver somniferum*	ECHo2450	modern		1896–1904	Fyssen Program
			Macrosample	Seeds	*Polygonum convolvulus*	ECHo2265	6240	30	5306–5067	Fyssen Program
Těšetice-Kyjovice	Czech Republic	Early Neolithic-LBK	Microsample	Seeds	*Papaver somniferum*	ECHo2449	5920	90	5010–4549	Fyssen Program
			Macrosample	Seeds	Cerealia	ECHo2656	6270	30	5318–5084	Fyssen Program
Isolino Virginia	Italy	Early Neolithic-Facies Isolino	Microsample	Seeds	*Papaver somniferum*	ECHo2451	5610	150	4796–4057	Fyssen Program
			Macrosample	Seeds	Cerealia	LTL2895A	5888	60	4932–4606	^35^
La Gillière 2	Switzerland	Early Neolithic-Néolithique ancien valaisan	Microsample	Seeds	*Papaver somniferum*	ECHo2452	5985	50	4999–4726	Fyssen Program
			Macrosample	Seeds	*Hordeum* sp.	ECHo2261	6070	30	5201–4849	Fyssen Program
Le Chenet des Pierres	France	Middle Néolithique VBQ/Saint-Uze	Microsample	Seeds	*Papaver somniferum*	ECHo2445	5370	60	4338–4050	Fyssen Program

A total of 20 samples were therefore studied, including 12 microsamples and 8 macrosamples that had been processed during the research program “Origin and early dispersal of the opium poppy in Europe during the Neolithic” funded by the Fyssen Fondation (2018–2019). In addition, two dates on macrosamples previously performed were taken into account at La Draga and Isolino Virginia.

## Results

The radiocarbon age of the microsamples are comprised between 6600 ± 50 BP and the modern period. The macrosamples gave a radiocarbon age comprised between 6295 ± 30 BP and 5888 ± 60 BP (Table 1). The two dates (ECHo2454 and ECHo2657) on micro- and macrosamples obtained from La Marmotta from two different archaeological contexts gave very similar results (Table 1). At Le Taï, La Draga, Remicourt-Fond de Momalle, Isolino Virginia and La Gillière, there was no significant difference in the ^14^C age between the micro- and the macrosamples (t-value < 5%), which indicates that the opium poppy is not intrusive (See Supplementary Information S3). At Buchères and Těšetice, there was a difference in the ^14^C age between the micro- and the macrosamples. At Buchères, the t-value of the combined dates was just above 5%. With a 2-sigma precision, the calibrated age of the opium poppy was slightly more recent than the age given by the macrosample (Table 1). The date of the microsample (ECHo2890) encompasses the end of the BVSG culture (i.e. the end of the Early Neolithic) and the Middle Neolithic I (Cerny culture). The pit in which the opium poppy was identified was attributed to the Early Neolithic/Cerny based on a ^14^C date, from the bottom of the pit (BVSG), and on ceramic fragments (Cerny), although the latter were poorly diagnosed^38^ (See Supplementary Information S2). The hundred opium poppy seeds could therefore be intrusive or the pit chronologically slightly later than the Early Neolithic. At Těšetice, the t-value is clearly above the 5% threshold; therefore, an intrusion of the microsample may be suspected. This is a multiperiod site with structures from the Neolithic period, the Bronze Age, and the Iron Age^39^. Nevertheless, at Buchères (ECHo2890) and Těšetice (ECHo2449), the opium poppies are attributed to the Neolithic period, allowing the two microsample dates to be included in the discussion on plant dispersal. Finally, at Le Chenet des Pierres, a sole opium poppy has been dated (ECHo2445). The margin of error is quite small (± 60 yr) compared to the other microsamples, enabling a rather accurate dating of opium poppy at this site, despite the absence of a macrosample.

At Los Castillejos, the ^14^C date from the macrosample (ECHo2260) was consistent with the expected chronology (i.e. Early Neolithic), but the direct date from the opium poppy seeds (ECHo 2443) was slightly younger. The site records an archaeological sequence comprised between the Early Neolithic to the end of Chalcolithic period^40^, so the microsample might be intrusive. At Smólsk 4, the opium poppy seed (ECHo 2450) was modern. There was some doubt regarding its preservation state (i.e. charred or desiccated) during the archaeobotanical analysis, but direct dating of the seed confirms that it is a modern intrusion (Table 1). An additional fragmented charred opium poppy seed was discovered at Smólsk 4 but was removed from the exploratory dating program because it was deemed too small for radiocarbon dating.

**Figure 3:**
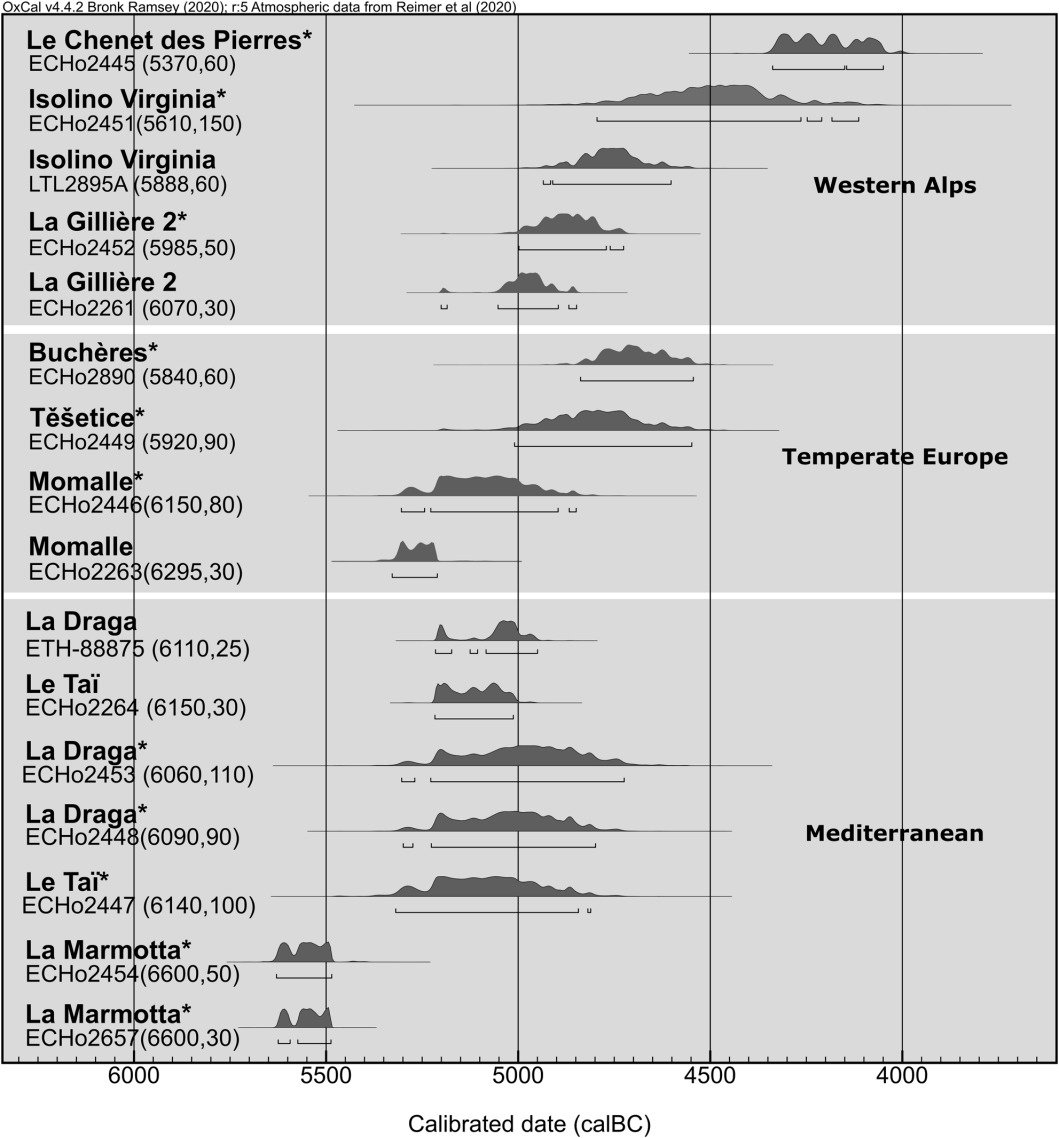
Chronology of the opium poppy in western Europe. The dates were calibrated with OxCal 4.4. software based on the IntCal20 atmospheric curve^59,60^. The asterisk corresponds to the dating performed directly on the opium poppy.

Calibration of the ^14^C dates provides a chronological distribution range (2- sigma) from 5622 to 4050 cal BCE (Fig. 3). The opium poppy from La Marmotta (central Italy) is dated to 5610–5480 cal BCE. In the Mediterranean, the following landmarks, from Le Taï (south of France) and La Draga (northeastern Spain), both comprise between 5311 and 4717 cal BCE. The dates from temperate Europe are between 5329 and 4546 cal BCE. Only the two dates from Momalle are attributed to the Linearbandkeramik period. Finally, in the western Alps, the chronological points are distributed between 5201 and 4050 cal BCE.

## Discussion

### Dating the Mediterranean Origin of Opium Poppy

Our work attests the antiquity of the capsules from La Marmotta (central Italy), dated to ca. 5620–5480 cal BCE (Table 1, Fig. 3), corresponding probably to the early stage of the site’s occupation, with regard the chronological information in our possession for this site^25^. The landmarks on short-lived taxa from the Epicardial Le Taï (south of France) and the Late Cardial La Draga (northeastern Spain) are both comprised between ca. 5200 and 5000 cal BCE. There is a 300 year gap between La Marmotta and Le Taï/La Draga, that may be due to the scarcity of pre-5200 cal BCE sites, including waterlogged pile-dwellings that offer optimal plant macroremains preservation. La Marmotta, Le Taï, and La Draga are all included in the current natural distribution area of the putative wild opium poppy^41^. The plant could therefore have grown naturally in specific spots along the central and western Mediterranean coasts and have been grown by the pioneer Neolithic farmers. The opium poppy seed (undated directly) identified at Peiro Signado, dated from the beginning of the six millennium^23^, could suggest the early integration of the crop in the Neolithic economy, as well as the presence of several areas of potential cultivation in the central and western Mediterranean Basin.

### Dating the Diffusion to Northwestern Temperate Europe

In northwestern temperate Europe, Momalle’s opium poppy (central Belgium) falls between 5300 and 4800 cal BCE, while the macrosample is at the very beginning of this range between 5300 and 5200 cal BCE. For this last sample, the ageing of the date performed on charcoal is questionable (see Sampling). Nevertheless, this range is consistent with the LBK regional occupation (ca. 5200- 5000 cal BCE)^42^. Furthermore, according to typo-chronology of ceramics and lithic raw material, the site of Momalle (sector III) would be linked to a pioneer phase of settlement in central Belgium^43^. Likewise, few undated charred opium poppy seeds have been found in other nearby pioneer LBK structures at Remicourt-En Bia Flo II and Waremme-Vinâve^10^. Similarly, charred seeds were discovered in structures dated to the Flomborn phase at Vaihingen an der Enz and Nieder-Mörlen (LBK II, ca. 5350–5200 cal BCE), both located in Germany, as well as at Geleen-Janskamperveld (ca. 5200–5000 cal BCE) in the Netherlands^27,29,44,45^. On this latter site, as at Momalle, the opium poppy is present since the genesis of the LBK regional occupation, as there is no LBK I in the Dutch Limburg^29^. Our study confirms that the opium poppy is an early addition to the Early Neolithic crop package west of the Rhine, probably at the latest from 5300–5200 cal BCE. Based on our dataset, we did not find any evidence for the presence of the opium poppy in the eastern LBK region. The microsample date from the multiperiod Těšetice (Czech Republic) is inconclusive due to the uncertainty in the calibrated range, spanning almost 500 years from 5016 to 4553 cal BCE. The microsample from Buchères gives the earliest securely chronological landmarks in the northern half of France and attests the presence of the plant between 4835 and 4545 cal BCE.

Radiocarbon dates are consistent with the hypothesis of opium poppy introduction in northwestern temperate Europe via Mediterranean farmers, who may have been using opium poppies for several centuries. The first direct contacts between the Cardial and the LBK populations are attested by ceramics, lithic tools or stone bracelets from ca. 5300–5200 BCE^46,47^. The rapidity of opium poppy dispersal, from the south to the north, could be explained by the ecology of the species, which currently adapts easily to a broad range of soil types and climatic tolerances, enabling it to colonize almost every environment, including loessic soils in temperate areas^48^. This new plant, therefore, was seemingly able to rapidly disperse across the whole LBK area (i.e. towards the east of the Rhine), possibly favored by intracultural exchange networks. At Vaihingen an der Enz, the opium poppy seems to be attached to one of the groups—characterized by differences in ceramic decorations and the lithic industry, for example—identified at the site^44^. This distribution could testify to a particular know-how, since the Early Neolithic, related to this crop that has several crucial uses for human societies (i.e. food, the oil contained in its seed, the psychoactive properties of the latex exuded by the capsules).

Paradoxically, naked cereals, commonly identified at Mediterranean Early Neolithic sites, are found occasionally in temperate Europe in recent LBK structures^10,22^. This hypothesis could testify to the continuity of the exchange networks between the western Mediterranean and the northwestern temperate Europe during the last third of the sixth millennium BCE.

### Dating the Diffusion to the Western Alps

The earliest attestation of opium poppy in the western Alps is dated to between 5000 and 4850 cal BCE at La Gillière 2. Another nearby Early Neolithic site, Tourbillon, delivered undated opium poppy seeds in very small quantities. On the southern slope of the Alps, the opium poppy of Isolino Virginia is dated to 4850–4550 cal BCE and is thus slightly more recent than La Gillière. In the northern French Alps, the plant is still present around 4300–4050 cal BCE at Le Chenet des Pierres. Regarding the connection between the Italian Alpine foothills and the upper Rhone Valley, it can be assumed that the plant was introduced through contacts with southern farmers around 5000–4800 BCE. On the other hand, the connection between the Alpine populations and the late LBK communities cannot be excluded as an explanation for the dispersal of the species in some areas of the western Alps. The opium poppy could therefore reflect a secondary acquisition via the Rhine corridor. One of the reasons for the late introduction of the crop, and of farming practices in general, in the western Alps is that hunter–gatherer populations may have been present until at least the first half of the fifth millennium^49^. The crop became widespread in the Alps, even in mid-altitude, from as early as the second half of the fifth millennium. In fact, the many pile-dwelling sites on the Swiss Plateau, dated between 4300 and 2600 cal BCE, have delivered the largest quantities of opium poppy remains known in Europe^50,51^.

## Conclusion

This first solid chrono-geographical framework of the early history of the opium poppy makes it possible to revisit the hypothesis on its diffusion from ca. 5600 to 4000 cal BCE in western Europe. Its presence is attested in central Italy from the middle of the sixth millennium BCE. The northward dispersal of the plant outside its native area started ca. 5300–5200 cal BCE. Its later introduction in the western Alps is attested ca. 5000–4800 cal BCE and reveals different dynamics of spread that may be due to the delayed expansion of the Neolithic in mountainous areas. The opium poppy therefore constitutes a relevant marker to discuss the complex phenomenon of the Neolithic genesis, movements of human populations, and inter-cultural relations from the beginning of the sixth millennium BCE in Europe.

Our work confirms the necessity of directly dating plant macroremains, in particular minute plant samples, to assess the chronology of their diffusion. It has highlighted intrusions of opium poppy seeds from more recent archaeological levels at Los Castillejos, Těšetice, and Buchères, or from the modern period at Smólsk. This research opens the way for the direct dating of small short-lived botanical samples. The low number of charred seeds, which is a common phenomenon in archaeological sites preserved in dry environments, does not prevent the dating of a large series of Neolithic sites.

This research is intended to be completed with additional chronological landmarks, in order to have a more detailed view of opium poppy dispersal in western Europe, adjacent European regions, and the Near East. Furthermore, this research needs to be supported by studies on the process of opium poppy domestication in the Early Neolithic, involving the use of geometric morphometrics on seed remains (A. Jesus, on-going PhD at the University of Basel), helping to clarify whether opium poppy was cultivated and domesticated in the Mediterranean or outside its natural area of distribution.

## Method

Due to their diminutive size and fragility, microsamples were only subjected to a gentle acid wash. They were then combusted offline using a manual line dedicated to very small samples. The amount of carbon was then estimated and the CO_2_ gas was split, when possible, prior to sealing in one or two glass tubes. The CO_2_ samples were then introduced directly into the AMS using the gas source interface system (GIS)^52^. Macrosamples were prepared using the classical acid–alkali–acid (AAA) treatment, then combusted and graphitized using an AGE 3 device (Ionplus, Switzerland)^53^. In order to reduce the risk of memory effects in the graphite reactors, a sample of similar age was combusted prior to each archaeological sample. All the samples were dated using the compact AMS ECHoMICADAS (i.e. Environment, Climate, Human MIni CArbon DAting System)^54^. For macrosamples, data reduction was performed using Bayesian Analysis of Time Series (BATS) software (version 4.07)^55^. Oxalic acid II NIST standard and phthalic anhydride blanks were measured, for each individual run, to allow normalization, correction for fractionation, and background corrections. For small samples we added a constant contamination correction (Mc = 0.3µgC and Rc = 0.64 F14C)^56^. The radiocarbon ages were calibrated using OxCal 4.4. software^57,58^ based on the IntCal 20 atmospheric curve.

## Supplementary information

Supplementary informations.
