# Supplementary material for: Direct dating reveals the early history of opium poppy in western Europe
Source: Sci Rep. 2020 Nov 20;10:20263. doi: 10.1038/s41598-020-76924-3 (PMC7679390; doi:10.1038/s41598-020-76924-3)
Supplement: Supplementary file 1 — Supplementary informations. [file 41598_2020_76924_MOESM1_ESM.docx]

**Direct dating reveals the early history of opium poppy in Western Europe**

Aurélie Salavert, Antoine Zazzo, Lucie Martin, Ferran Antolín, Caroline Gauthier, François Thil, Olivier Tombret, Laurent Bouby, Claire Manen, Mario Mineo, Aldona Mueller-Bieniek, Raquel Piqué, Rottoli Mauro, Núria Rovira, Françoise Toulemonde, Ivana Vostrovská.

**Index**

**Supplementary File S1.** Inventory of the Early Neolithic sites with opium poppies based on the literature reviews. For location of the sites see Fig. 1 and the online open access map: <http://umap.openstreetmap.fr/fr/map/salavertetal_sr_fig12_460185>

**Supplementary File S2.** Presentation of the archaeological sites and the samples included to the paper.

**Supplementary File S3.** In sites where micro and macrosamples were dated, a Chi-square test validity test with an acceptance threshold of 5% was applied to evaluate if the microsample might be intrusive.

**References**

**Supplementary File S1.** Inventory of the Early Neolithic sites with opium poppies based on the literature reviews. For location of the sites see Fig. 1 and the online open access map: <http://umap.openstreetmap.fr/fr/map/salavertetal_sr_fig12_460185>

| **Zone** | **Site** | **Country** | **Chronoculture of the site** | **References** |
| --- | --- | --- | --- | --- |
| Near-East | Atlit-Yam | Israel | PPNC | ^1^ |
|  | Körtik-Tepe | Turkey | Aceramic Neolithic | ^2^ |
| Mediterranean | Camp del Colomer | Andorra | Late epi-Cardial | ^3^ |
|  | Cueva de Los Murciélagos | Spain | Early Neolithic | ^4^ |
|  | La Draga 7a (DG-VIIa) | Spain | Late Cardial | ^3^ |
|  | La Lámpara | Spain | Early Neolithic | ^5^ |
|  | La Marmotta | Italy | Impressa | ^6^ |
|  | Los Castillejos Fase 3 | Spain | Epi-Cardial | ^7^ |
|  | Peiro Signado | France | Impressa | ^8^ |
|  | Remoulins-Le Taï | France | Epi-Cardial | ^9^ |
| Temperate Europe | Alleur | Belgium | LBK | ^10^ |
|  | Aubechies-Coron Maton | Belgium | LBK | ^11^ |
|  | Beek-Kerkeveld | Netherlands | LBK | ^12^ |
|  | Bréviandes-ZAC Saint-Martin | France | LBK | ^13^ |
|  | Brodau | Germany | LBK | ^14^ |
|  | Bruchenbrücken | Germany | LBK | ^15^ |
|  | Eggingen | Germany | LBK | ^16^ |
|  | Erkelenz-Kuckhoven | Germany | LBK | ^17^ |
|  | Eythra | Germany | LBK | ^18^ |
|  | Fauerbach vor der Höhe | Germany | LBK | ^19^ |
|  | Fechenheim | Germany | LBK | ^19^ |
|  | Garsdorf | Germany | LBK | ^20^ |
|  | Geleen-Haesselderveld | Netherland | LBK | ^21^ |
|  | Buchères-Parc Logistique de l’Aube 2013 | France | BVSG | ^22^ |
|  | Geleen-Janskamperveld | Netherland | LBK (Flomborn) | ^23^ |
|  | Herxheim/Landau | Germany | LBK | ^19^ |
|  | Langweiler 2 | Germany | LBK | ^24^ |
|  | Langweiler 6 | Germany | LBK | ^25^ |
|  | Langweiler 8 | Germany | LBK | ^26^ |
|  | Langweiler 9 | Germany | LBK | ^27^ |
|  | Laurenzberg 8 | Germany | LBK | ^28^ |
|  | Leipzig-Plaußig | Germany | LBK | ^18^ |
|  | Meindling | Germany | LBK | ^29^ |
|  | Mittelbuchen | Germany | LBK | ^19^ |
|  | Nieder-Mörlen | Germany | LBK (Flomborn) | ^19^ |
|  | Oekoven | Germany | LBK | ^20^ |
|  | Oleye-Al Zepe | Belgium | LBK | ^30^ |
|  | Remicourt-En-Bia Flo II | Belgium | LBK | ^31^ |
|  | Remicourt-Fond de Momalle | Belgium | LBK | ^31^ |
|  | Rosdorf-Mühlengrund | Germany | LBK | ^32^ |
|  | Schkeuditz-Altscherbitz | Germany | LBK | ^18^ |
|  | Smólsk 4 | Poland | LBK | ^33^ |
|  | Těšetice-Kyjovice | Czech Republic | LBK | ^34^ |
|  | Usingen | Germany | LBK | ^19^ |
|  | Vaihingen an der Enz | Germany | LBK (Flomborn) | ^35^ |
|  | Vaux-et-Borset et Gibour | Belgium | LBK | ^36^ |
|  | Wanlo | Germany | LBK | ^28^ |
|  | Waremme-Vinave | Belgium | LBK | ^30^ |
| western Alps | Tourbillon | Switzerland | Early Neolithic/Middle Neolithic I (transition) | ^37^ |
|  | La Gillière 2 (F216) | Switzerland | Early Neolithic | ^37^ |

**Supplementary File S2.** Presentation of the archaeological sites and the samples included to the paper.

# **Buchères-les Terriers, Parc Logistique de l'Aube 2013 (Temperate Europe)**

| **GENERAL ARCHAEOLOGICAL CONTEXT** | | |
| --- | --- | --- |
| **Country** | France | |
| **Province/district, region** | Grand-Est | |
| **Town** | Buchères | |
| **Altitude (a.s.l.)** | 120 m | |
| **Type of site** | Open air settlement | |
| **Cultural attribution(s) of the site** | Early Neolithic | |
| **Type of layer/structure** | Pit | |
| **Cultural attribution of the structure** | Early Neolithic (VSG)/Middle Neolithic I (Cerny) | |
| **Existing radiocarbon dates** | 5930 ± 35 BP (Poz-60673), D40F0108, US 6, charcoal | |
| **Archaeologist.s (institution)** | Paresys C. (Institut National de Recherches Archéologiques Préventives, INRAP) | |
| **Main publication(s)** | ^38^ | |
| **SAMPLE DESCRIPTION** | | |
| **Type of sample** | Macrosample | Microsample |
| **Sample denomination** | Fosse D400108 us7-us8 | Fosse D400108 us7-us8 |
| **Date of sampling** | 2013 | 2013 |
| **Type of macroremain** | Cotyledon | Seeds |
| **Taxa** | *Pisum sativum* | *Papaver somniferum* |
| **Number of specimens submitted for dating** | 2 | 15 |
| **Total number of specimens** | - | 15 |
| **Preservation** **and type of remain** | Charred | Charred |
| **Archaeobotanist** | Toulemonde F. | |
| **Main publication(s) of the macro-remains analyses** | ^22^ | |

**Isolino Virginia (western Alps)**

| **GENERAL ARCHAEOLOGICAL CONTEXT** | |
| --- | --- |
| **Country** | Italy |
| **Province/district, region** | Varese, Lombardia |
| **Town** | Biandronno |
| **Altitude (a.s.l.)** | 330 m |
| **Type of site** | Pile-dwelling |
| **Cultural attribution(s) of the site** | Early Neolithic |
| **Type of layer/structure** | Occupation layer |
| **Cultural attribution of the structure** | Facies Isolino |
| **Existing radiocarbon dates** | 5888 ± 60 BP (LTL 2895A), US 63, charred seeds of cereals |
| **Archaeologist.s (institution)** | Giuseppina Banchieri D. (formerly Musei Civici di Villa Mirabello, Varese, Italy) |
| **Main publication(s)** | ^39^ |
| **SAMPLE DESCRIPTION** | |
| **Type of sample** | Microsample |
| **Sample denomination** | Isolino VA US 63 |
| **Date of sampling** | 2006 |
| **Type of macroremain** | Seeds |
| **Taxa** | *Papaver somniferum* |
| **Number of specimens submitted for dating** | 12 |
| **Total number of specimens** | 65 |
| **Preservation** **and type of remain** | Originally waterlogged, dried after sieving |
| **Archaeobotanist** | Rottoli M. |
| **Main publication(s) of the macro-remains analyses** | ^6^ |
| **Additional information** |  |

**La Draga (Mediterranean)**

| **GENERAL ARCHAEOLOGICAL CONTEXT** | | |
| --- | --- | --- |
| **Country** | Spain | |
| **Province/district, region** | Catalunya | |
| **Town** | Banyoles | |
| **Altitude (a.s.l.)** | 172 m | |
| **Type of site** | Pile-dwelling | |
| **Cultural attribution(s) of the site** | Early Neolithic | |
| **Cultural attribution of the structure** | Late Cardial (5300-5000 BCE) | |
| **Existing radiocarbon dates** | 6121 ± 33 BP (OxA-20232), JH-84-85 (level II)  6110 +/- 26 BP (ETH-88875), JD/JE78 | |
| **Archaeologist.s (institution)** | Piqué R. (Universitat Autònoma de Barcelona) | |
| **Main publication(s)** | ^40^ | |
| **SAMPLE DESCRIPTION** | | |
| **Type of sample** | Microsample | Microsample |
| **Sample denomination** | DG97-JH84-85-II | DG11-JD/JE78-7001 |
| **Date of sampling** | 1997 | 2011 |
| **Type of layer/structure** | Occupation layer | Pit |
| **Type of macroremain** | Seeds | Seeds |
| **Taxa** | *Papaver somniferum* | *Papaver somniferum* |
| **Number of specimens submitted for dating** | 6 | 6 |
| **Total number of specimens** | Ca. 60 | >100 |
| **Preservation** **and type of remain** | Originally charred and waterlogged, dried after sieving | Originally waterlogged, dried after sieving |
| **Archaeobotanist** | Antolín F. | |
| **Main publication** | ^41^ | |
| **Additional information** | The microsamples (ECHo2448.1.1 and ECHo2453.1.1) come from two different contexts of the level II. | |

**La Gillière 2 (western Alps)**

| **GENERAL ARCHAEOLOGICAL CONTEXT** | | |
| --- | --- | --- |
| **Country** | Switzerland | |
| **Province/district, region** | Valais | |
| **Town** | Sion | |
| **Altitude (a.s.l.)** | 500 m | |
| **Type of site** | Dry open-air habitat | |
| **Cultural attribution(s) of the site** | Early (“Néolithique ancient valaisan”)/Middle Neolithic | |
| **Type of layer/structure** | Hearth, F216 | |
| **Cultural attribution of the structure** | Early Neolithic | |
| **Existing radiocarbon dates** | 5973±31 BP (UBA-23154) | |
| **Archaeologist.s (institution)** | Baudais D. (University of Geneva, Switzerland) | |
| **Main publication(s)** | ^42^ | |
| **SAMPLE DESCRIPTION** | | |
| **Sample denomination** | SG93-F216-H4 | |
| **Date of sampling** | 1993 | |
| **Type of sample** | Microsample | Macrosample |
| **Type of macroremain** | Seeds | Seeds |
| **Taxa** | *Papaver somniferum* | *Hordeum* sp. |
| **Number of specimens submitted for dating** | 20 | 4 |
| **Total number of specimens** | 3188 | 464 |
| **Preservation** **and type of remain** | Charred | Charred |
| **Archaeobotanist** | Martin L. | |
| **Main publication** | ^37^ | |

**La Marmotta (Mediterranean)**

| **GENERAL ARCHAEOLOGICAL CONTEXT** | | |
| --- | --- | --- |
| **Country** | Italy | |
| **Province/district, region** | Roma, Lazio | |
| **Town** | Anguillara Sabazia (Lago di Bracciano) | |
| **Altitude (a.s.l.)** | 164 m | |
| **Type of site** | Pile-dwelling | |
| **Cultural attribution(s) of the site** | Early Neolithic | |
| **Type of layer/structure** | Occupation Layer | |
| **Cultural attribution of the structure** | Earliest phase: Cardial (Stile di Basi-Pienza); Later phase (ceramica lineare nello stile della facies Sasso –Fiorano) | |
| **Existing radiocarbon dates** | 6425 ± 21 BP (Hd 20411), wood  6474 ± 20 BP (Hd 20466), wood | |
| **Archaeologist.s (institution)** | Mineo M. (Museo delle Civiltà - Museo Preistorico Etnografico "Luigi Pigorini", Roma, Italy), Fugazzola Delpino M.A., formerly superintendent of Museo Nazionale Preistorico ed Etnografico “Luigi Pigorini”, Rome (now Museo delle Civiltà) | |
| **Main publication(s)** | ^43^ | |
| **SAMPLE DESCRIPTION** | | |
| **Type of sample** | Microsample | Macrosample |
| **Sample denomination** | AS22 [q. A184, Liv. 1 torb., n. 25431] | C25 [q. A134, spolvero piroga, n. 25936] |
| **Date of sampling** | 2001 | 2001 |
| **Type of macroremain** | Stigma (micro-sample) | Stigma (macro-sample) |
| **Taxa** | *Papaver somniferum* | *Papaver somniferum* |
| **Number of specimens submitted for dating** | 4 fragments | 2 |
| **Total number of specimens** | - | - |
| **Preservation** **and type of remain** | Charred | Charred |
| **Archaeobotanist** | Rottoli M. | |

**Le Chenet des Pierres (western Alps)**

| **GENERAL ARCHAEOLOGICAL CONTEXT** | |
| --- | --- |
| **Country** | France |
| **Province/district, region** | Savoie |
| **Town** | Bozel |
| **Altitude (a.s.l.)** | 950 m |
| **Type of site** | Dry open-air habitat |
| **Cultural attribution(s) of the site** | Middle Neolithic (4400-4000 cal BCE) |
| **Type of layer/structure** | Occupation layer |
| **Cultural attribution of the structure** | VBQ/Saint-Uze |
| **Existing radiocarbon dates** | 5375 ± 45 BP (Ly 2455) |
| **Archaeologist.s (institution)** | Rey P.-J. (UMR 5204, Edytem, University of Savoie Mont-Blanc) |
| **Main publication(s)** | ^44^ |
| **SAMPLE DESCRIPTION** | |
| **Type of sample** | Microsample |
| **Sample denomination** | BLMC.d392 |
| **Date of sampling** | 2005 |
| **Type of macroremain** | Seeds |
| **Taxa** | *Papaver somniferum* |
| **Number of specimens submitted for dating** | 12 |
| **Total number of specimens** | 421 |
| **Preservation** **and type of remain** | Charred |
| **Archaeobotanist** | Martin L. |
| **Main publication** | ^45^ |

**Los Castillejos - Las Peñas de los Gitanos (Mediterranean)**

| **GENERAL ARCHAEOLOGICAL CONTEXT** | | |
| --- | --- | --- |
| **Country** | Spain | |
| **Province/district, region** | Granada | |
| **Town** | Montefrio | |
| **Altitude (a.s.l.)** | 1028 m | |
| **Type of site** | Dry open-air site | |
| **Cultural attribution(s) of the site** | Early Neolithic (Cardial)-Middle Bronze Age (5200-2000 BCE) | |
| **Type of layer/structure** | Hearth (micro-sample) and anthropic layer (waste) located near the hearth (macro-sample). | |
| **Cultural attribution of the structure** | Early Neolithic (Cardial). Phase 3. | |
| **Existing radiocarbon dates** | 6120 ± 40 BP | |
| **Archaeologist.s (institution)** | Molina González F., Cámara Serrano J.A., Afonso Marrero J.A. (Departamento de Prehistoria, universidad de Granada). | |
| **Main publication(s)** | ^46,47^ | |
| **SAMPLE DESCRIPTION** | | |
| **Type of sample** | Microsample | Macrosample |
| **Sample denomination** | MF612785 | MF613868 |
| **Date of sampling** | 1994(?) | 1994(?) |
| **Type of macroremain** | Seeds | Seeds |
| **Taxa** | *Papaver somniferum* ssp. *setigerum* | *Hordeum vulgare* var. *nudum* |
| **Number of specimens submitted for dating** | 3 | 4 |
| **Total number of specimens** | 3 | 4 |
| **Preservation** **and type of remain** | Charred | Charred |
| **Archaeobotanist** | Rovira N. | |
| **Main publication** | ^7^ | |

**Remicourt-Fond de Momalle (Temperate Europe)**

| **GENERAL ARCHAEOLOGICAL CONTEXT** | | |
| --- | --- | --- |
| **Country** | Belgium | |
| **Province/district, region** | Hesbaye | |
| **Town** | Remicourt | |
| **Altitude (a.s.l.)** | 130 m | |
| **Type of site** | Dry open-air habitat | |
| **Cultural attribution(s) of the site** | Early Neolithic (Linearbandkeramik) | |
| **Type of layer/structure** | South longitudinal pit (F222) of the house I (sector III) | |
| **Cultural attribution of the structure** | Ancient and middle Linearbankeramik | |
| **Existing radiocarbon dates** | - | |
| **Archaeologist.s (institution)** | Fock H. (AWaP) | |
| **Main publication(s)** | ^48^ | |
| **SAMPLE DESCRIPTION** | | |
| **Sample denomination** | st.222-83cm-D | |
| **Date of sampling** | 1998 | |
| **Type of sample** | Microsample | Macrosample |
| **Type of macroremain** | Seeds | Charcoal |
| **Taxa** | *Papaver somniferum* | *Fraxinus* sp. |
| **Number of specimens submitted for dating** | 5 | - |
| **Total number of specimens** | 5 | - |
| **Preservation** **and type of remain** | Charred | Charred |
| **Archaeobotanist** | Salavert A. | |
| **Main publication** | ^31^ | |
| **Additional information** |  | |

**Remoulins-Le Taï (Mediterranean)**

| **GENERAL ARCHAEOLOGICAL CONTEXT** | | |
| --- | --- | --- |
| **Country** | France | |
| **Province/district, region** | Gard | |
| **Town** | Remoulins | |
| **Altitude (a.s.l.)** | 61 m | |
| **Type of site** | Dry open-air and cave habitat | |
| **Cultural attribution(s) of the site** | Early Neolithic to Late Neolithic | |
| **Type of layer/structure** | Pit | |
| **Cultural attribution of the structure** | Early Neolithic (Epicardial) | |
| **Existing radiocarbon dates** | Many dates between 5270 and 4990 cal BCE | |
| **Archaeologist.s (institution)** | Manen C. (CNRS) | |
| **Main publication(s)** | ^49^ | |
| **SAMPLE DESCRIPTION** | | |
| **Type of sample** | Microsample | Macrosample |
| **Sample denomination** | T04-FS043-N11a-d37-p056 | T11-FS043-M10b-d29-p175 |
| **Date of sampling** | 2004 | 2011 |
| **Type of macroremain** | Seeds | Seeds |
| **Taxa** | *Papaver somniferum* subsp. *setigerum/somniferum* | *Triticum* sp. |
| **Number of specimens submitted for dating** | 3 | 1 |
| **Total number of specimens** | 11 | 9 |
| **Preservation** **and type of remain** | Charred | Charred |
| **Archaeobotanist** | Bouby L. | |
| **Main publication** | ^9^ | |
| **Additional information** |  | |

**Smólsk 4 (Temperate Europe)**

| **GENERAL ARCHAEOLOGICAL CONTEXT** | | |
| --- | --- | --- |
| **Country** | Poland | |
| **Province/district, region** | Kuyavia (Kujawy) | |
| **Town** | Smólsk | |
| **Altitude (a.s.l.)** | 80 m | |
| **Type of site** | Dry open-air habitat | |
| **Cultural attribution(s) of the site** | Early Neolithic maybe multicultural | |
| **Type of layer/structure** | Clay pit 1 | |
| **Cultural attribution of the structure** | Linearbandkeramik | |
| **Existing radiocarbon dates** |  | |
| **Archaeologist.s (institution)** | Ryszard G.(Muzeum Archeologiczne i Etnograficzne w Łodzi) | |
| **Main publication(s)** |  | |
| **SAMPLE DESCRIPTION** | | |
| **Type of sample** | Microsample | Macrosample |
| **Sample denomination** | 2 | 3 |
| **Date of sampling** | 2002 | 2002 |
| **Type of macroremain** | Seeds | Seeds |
| **Taxa** | *Papaver* cf. *somniferum* | *Polygonum convolvulus* |
| **Number of specimens submitted for dating** | 1 | 3 |
| **Total number of specimens** | 1 | >30 |
| **Preservation** **and type of remain** | Probably not charred | Charred |
| **Archaeobotanist** | Mueller-Bieniek A. | |
| **Main publication** | ^33^ | |
| **Additional information** | The uncharred seed is not mentioned as probable modern contaminaton, the charred seed was fragmented and rejected from the radiocarbon dating programm | |

**Těšetice-Kyjovice (Temperate Europe)**

| **GENERAL ARCHAEOLOGICAL CONTEXT** | | |
| --- | --- | --- |
| **Country** | Czech Republic | |
| **Province/district, region** | Znojmo | |
| **Town** | Těšetice | |
| **Altitude (a.s.l.)** | 255 m | |
| **Type of site** | Dry open-air habitat | |
| **Cultural attribution(s) of the site** | Early Neolithic/Middle Neolithic/Bronze age/Iron age | |
| **Type of layer/structure** | Post-hole of LBK house (KJ1510) | |
| **Cultural attribution of the structure** | Linearbandkemarik (phase IIa) | |
| **Existing radiocarbon dates** | 6210 ± 40 BP (Poz-38793) | |
| **Archaeologist.s (institution)** | Vostrovská, I. | |
| **Main publication(s)** | ^50^ | |
| **SAMPLE DESCRIPTION** | | |
| **Type of sample** | Microsample | Macrosample |
| **Sample denomination** | Y6-5h-V225 | DG11-JD/JE78-7001 |
| **Date of sampling** | 2012 | 2011 |
| **Type of macroremain** | Seeds (micro-sample) | Seeds (macro-sample) |
| **Taxa** | *Papaver somniferum* | Cerealia |
| **Number of specimens submitted for dating** | 5 | 2 |
| **Total number of specimens** | 5 | 2 |
| **Preservation** **and type of remain** | Charred | Charred |
| **Archaeobotanist** | Lukšíková H. | |
| **Main publication** | ^34^ | |

**Supplementary File S3.** In sites where micro and macrosamples were dated, a Chi-square test validity test with an acceptance threshold of 5% was applied to evaluate if the microsample might be intrusive.

| **Site name** | **Country** | **Zone** | **Sample labels (micro/macro)** | **Chi 2 test** |
| --- | --- | --- | --- | --- |
| Remoulins-Le Taï | France | Mediterranean | ECHo2447.1.1/ ECHo2264.1.1 | df=1 T=0.0 (5% 3.8) |
| La Draga | Spain | Mediterranean | ECHo 2448.1.1/ ETH-88875 | df=1 T=0.0 (5% 3.8) |
| Isolino Virginia | Italy | Mediterranean | ECHo2451.1.1/ LTL2895A | df=1 T=2.9 (5% 3.8) |
| Remicourt - Fond de Momalle | Belgium | Temperate Europe | ECHo2446.1.1/ ECHo2263.1.1 | df=1 T=2.8 (5% 3.8) |
| Buchères- les Terriers | France | Temperate Europe | ECHo2890.1.1/ ECHo2262.1.1 | df=1 T=5.6 (5% 3.8) |
| Těšetice-Kyjovice | Czech Republic | Temperate Europe | ECHo2449.1.1/ ECHo2656.1.1 | df=1 T=13.1 (5% 3.8) |
| La Gillière 2 | Switzerland | Western Alps | ECHo2452.1.1/ ECHo2261.1.1 | df=1 T=2.1 (5% 3.8) |

**References**

1. Kislev, M. E., Hartmann, A. & Galili, E. Archaeobotanical and archaeoentomological evidence from a well at Atlit-Yam indicates colder, more humid climate on the Israeli coast during the PPNC period. *Journal of Archaeological Science* **31**, 1301–1310 (2004).

2. Rössner, C., Deckers, K., Benz, M., Özkaya, V. & Riehl, S. Subsistence strategies and vegetation development at Aceramic Neolithic Körtik Tepe, southeastern Anatolia, Turkey. *Vegetation History and Archaeobotany* **27**, 15–29 (2018).

3. Antolín, F. *Local, intensive and diverse?: Early farmers and plant economy in the North-East of the Iberian Peninsula (5500-2300 cal BC)*. vol. 2 (Barkhuis, 2016).

4. Pena-Chocarro, L. Early agriculture in central and southern Spain. *The origins and spread of domestic plants in Southwest Asia and Europe* 173–187 (2007).

5. Stika, H.-P. Early Neolithic agriculture in Ambrona, Provincia Soria, central Spain. *Vegetation History and Archaeobotany* **14**, 189–197 (2005).

6. Banchieri, D. & Rottoli, M. *Una nuova data per la storia del papavero da oppio (Papaver somniferum subsp. somniferum)*. vol. XXV (Sibrium, 2004).

7. Rovira Buendía, N. Agricultura y gestión de los recursos vegetales en el sureste de la península ibérica durante la prehistoria reciente. (Universitat Pompeu Fabra. Institut Universitari d’Història Jaume Vicens i Vives, 2007).

8. Bouby, L. *et al.* Early Neolithic (ca. 5850-4500 cal BC) agricultural diffusion in the Western Mediterranean: An update of archaeobotanical data in SW France. *PLOS ONE* **15**, e0230731 (2020).

9. Bouby, L., Durand, F., Rousselet, O. & Manen, C. Early farming economy in Mediterranean France: fruit and seed remains from the Early to Late Neolithic levels of the site of Taï (ca 5300–3500 cal bc). *Veget Hist Archaeobot* **28**, 17–34 (2019).

10. Dietsch-Sellami, M.-F. Étude carpologique sur le site rubané d’Alleur-À l’Arbre de la Mère Dieu (Province de Liège, Belgique). *Notae Praehistoricae* **24**, 129–133 (2004).

11. Constantin, C. *et al.* Le site rubané d’Aubechies ‘Coron Maton’ (Hainaut): Les fouilles de 2012. *Bulletin du Cercle archéologique Hesbaye-Condroz* **XXXIII**, 7–36 (2018).

12. Bakels, C. C. *Four linearbandkeramik settlements and their environment: a pal...* (Leiden University Press, 1978).

13. Bonnaire, E. 3.4. Les macrorestes végétaux. in *Bréviandes ZAC Saint-Martin 1. Un village de la colonisation danubienne initiale à forte composante ‘non rubanée’. Occupations mésolithiques, sépultures collectives Néolithique final, nécropole Bronze ancien/moyen, habitat RSFO/Hallstatt C.* (2017).

14. Herbig, C. Unkraut oder in Gärten kultivierte Heilpflanze? Die Rolle des Schwarzen Bilsenkrauts (Hyoscyamus niger L.) im Neolithikum–Neue archäobotanische Nachweise in linienbandkeramischen Brunnenbefunden in Sachsen [Weed or Medical Plant Cultivated in Gardens? The Role of Black Henbane (Hyoscyamus niger L.) in the Neolithic]. *Frankfurter archäologische Schriften* **18**, 147–157 (2012).

15. Kreuz, A. Archaeobotanical perspectives on the beginning of agriculture north of the Alps. in *The origins and spread of domestic plants in southwest Asia and Europe* (eds. Colledge, S. & Conolly, J.) 259–294 (2007).

16. Gregg, S. A. Paleo-ethnobotany of the Bandkeramik phases. in *Ulm-Eggingen: Die Ausgrabungen1982 bis 1985 in der bandkeramischen Siedlung und der mittelalterlichen W stung.* (ed. Kind, C.-J.) 367–399 (Konrad eiss verlag, 1982).

17. Knörzer, K.-H. Botanische Untersuchungen am bandkeramischen Brunnen von Erkelenz-Kückhoven. in *Brunnen der Jungsteinzeit.* (ed. Koschik, H.) 229–246 (Rheinland-Verlag, 1998).

18. Herbig, C., Maier, U., Stäuble, H. & Elburg, R. „Neolithische Füllhörner“ Archäobotanische Untersuchungen in fünf linienbandkeramischen Brunnen in Westsachsen. *Offa* **69/70**, 265–293 (2012).

19. Kreuz, A., Marinova, E., Schäfer, E. & Wiethold, J. A comparison of early Neolithic crop and weed assemblages from the Linearbandkeramik and the Bulgarian Neolithic cultures: differences and similarities. *Vegetation History and Archaeobotany* **14**, 237–258 (2005).

20. Knörzer, K. H. Urgeschichtliche Unkräuter im Rheinland ein beitrag zur Entstehungsgeschichte der Segetalgesellschaften. *Vegetatio* **23**, 89–111 (1971).

21. Bakels, C. C. & Rousselle, R. Restes botaniques et agriculture du Néolithique ancien en Belgique et aux Pays-Bas. *Helinium Wetteren* **25**, 37–57 (1985).

22. Toulemonde, F. *et al.* A brief history of plants in north-eastern France: 6,000 years of crop introduction in the Plain of Troyes, Champagne. *Vegetation History and Archaeobotany* (accepted).

23. Bakels, C. Aspects of crops and crop processing in the Linear-bandkeramik settlement of Geleen-Janskamperveld, The Netherlands. in *Excavations at Geleen-Janskamperveld 1990/1991* (ed. van de Velde, P.) 91–97 (Faculty of Archaeology, Leiden University, 2007).

24. Knörzer, K. H. Botanisches Material. in *Der bandkeramische Siedlungsplatz Langweiler 2, Gemeinde Aldenhoven, Kreis Düren: Gemeinde Aldenhoven, Kreis Düren* (ed. Farruggia, J.-P.) 139–152 (Rheinland-Verlag, 1973).

25. Knörzer, K. H. Subfossile Pflanzenreste aus der bandkeramischen Siedlung Langweiler 3 und 6, Kreis Jülich, und ein urnenfelderzeitlicher Getreidefund innerhalb dieser Siedlung. *Bonner Jahrbücher* **172**, 395–403 (1972).

26. Knörzer, K.-H. Untersuchungen der Früchte und Samen. in *Der bandkeramische Siedlungsplatz Langweiler 8. Gemeinde Aldenhoven, Kreis Düren.* (eds. Boelicke, U., Von Brandt, D., Lüning, J., Stelhi, P. & Zimmerman, A.) vol. 8 813–852 (1988).

27. Knörzer, K. H. Pflanzliche Grossreste des bandkeramischen Siedlungsplatzes Langweiler 9. *Rheinische Ausgrabungen* **18**, 279–303 (1977).

28. Knörzer, K. H. Subfossile Pflanzenreste aus der jüngerlatène-zeitlichen Siedlung bei Laurenzberg, Gem. Eschweiler, Kr. Aachen. *Bonner Jahrbücher des Rheinischen Landesmuseums in Bonn Bonn* 442–457 (1980).

29. Bakels, C. C. Fruits and seeds from the Linearbandkeramik settlement at Meindling, Germany, with special reference to Papaver somniferum. *Analecta Praehistorica Leidensia* **25**, 55–68 (1996).

30. Jadin, I. & Heim, J. Sur la voie de l’orge et du pavot: macrorestes végétaux et agriculture rubanée du Haut Geer dans un cadre européen. in *Trois petits tours et puis s’ en vont… La fin de la présence danubienne en Moyenne-Belgique.* vol. 109 345–392 (ERAUL, 2003).

31. Salavert, A. Plant economy of the first farmers of central Belgium (Linearbandkeramik, 5200–5000 b.c.). *Veget Hist Archaeobot* **20**, 321–332 (2011).

32. Kirleis, W. & Willerding, U. Die pflanzenreste aus der linienbandkeramischen Siedlung von Rosdorf-Mühlengrund, Ldkr. Göttingen, im südöstlichen Niedersachsen. *Praehistorische Zeitschrift* **83**, 133–178 (2008).

33. Bieniek, A. Neolithic plant husbandry in the Kujawy region of central Poland. *The origins and spread of domestic plants in Southwest Asia and Europe* **1**, 327–342 (2007).

34. Vostrovská, I., Bíšková, J., Lukšíková, H., Kočár, P. & Kočárová, R. The Environment and Subsistence of the Early Neolithic Settlement Area at Těšetice-Kyjovice, Czech Republic. *Environmental Archaeology* **23**, 248–262 (2019).

35. Bogaard, A. *Plant Use and Crop Husbandry in an Early Neolithic Village: Vaihingen an der Enz, Baden-Württemberg*. (2012).

36. Heim, J. & Hauzeur, A. Paysage paléobotanique des sites du Rubané et du groupe de Blicquy à Vaux-et-Borset" Gibour"(Hesbaye, Belgique). Culture du blé nu et récolte de pommes en contexte blicquien. *Bulletin de la Société préhistorique française* 289–305 (2002).

37. Martin, L. Plant economy and territory exploitation in the Alps during the Neolithic (5000–4200 cal bc): first results of archaeobotanical studies in the Valais (Switzerland). *Veget Hist Archaeobot* **24**, 63–73 (2015).

38. Paresys, C. *Buchères, Aube, «les Terriers» (Parc Logistique de l’Aube). Parc Logistique de l’Aube; l’évolution d’un terroir dans la plaine de Troyes (V: campagnes de fouille 2012-2013)*. (2019).

39. Banchieri, D. G. Isolino Virginia-Lago di Varese: archeologia e ambiente. *Il palù di livenza e le palafitte del sito unesco: nuovi studi e ricerche* 41–45 (2017).

40. Palomo, A. *et al.* Prehistoric occupation of Banyoles lakeshore: results of recent excavations at La Draga site, Girona, Spain. *Journal of Wetland Archaeology* **14**, 58–73 (2014).

41. Antolín, F., Buxó, R., Jacomet, S., Navarrete, V. & Saña, M. An integrated perspective on farming in the early Neolithic lakeshore site of La Draga (Banyoles, Spain). *Environmental Archaeology* **19**, 241–255 (2014).

42. Baudais, D. *Le site archéologique de la Gillière 2: Sion-Valais: rapport de fouille et d’élaboration intermédiaire 1993*. (Université de Genève-Département d’anthropologie et d’écologie, 1994).

43. Fugazzola Delphino, M. A. F. Dati di cronologia da un villaggio del Neolitico Antico. Le indagini dendrocronologiche condotte sui legni de La Marmotta (lago di Bracciano-Roma. *in Miscellanea in ricordo di Francesco Nicosia, Studia Erudita, Fabrizio Serra Editore* 1–10 (2010).

44. Rey, P.-J. Le site du Chenet des Pierres aux Moulins de Bozel (Savoie, France): une nouvelle séquence néolithique alpine. in *Alpis Graia Archéologie sans frontières autour du col du Petit-Saint-Bernard* 361–370 (2006).

45. Martin, L., Jacomet, S. & Thiebault, S. Plant economy during the Neolithic in a mountain context: the case of “Le Chenet des Pierres” in the French Alps (Bozel-Savoie, France). *Vegetation history and archaeobotany* **17**, 113–122 (2008).

46. Camara Serrano, J. A., Molina González, F. & Afonso Marrero, J. A. *La cronología absoluta de Los Castillejos en Las Peñas de los Gitanos (Montefrío, Granada)*. (Universidad de Cantabria, 2005).

47. Cámara Serrano, J. A., Afonso Marrero, J. A. & Molina González, F. La ocupación de las Peñas de los Gitanos (Montefrío, Granada) desde el Neolítico al mundo romano. Asentamiento y ritual funerario. in *Arqueología e historia de un paisaje singular: La Peña de los Gitanos, Montefrío (Granada)* (ed. Pedregosa, R.) 17–121 (Ayuntamiento de Montefrío, 2016).

48. Fock, H., Goffioul, C. & Cornélusse, F. Fouille d’un habitat rubané à Remicourt, au lieu-dit Fond de Momalle, secteur III. *Notae Praehistoricae* 123–129 (1998).

49. Caro, J. & Manen, C. Les productions céramiques du Néolithique ancien du Taï (Remoulins, Gard). Approche spatiale, caractérisation typo-technologique et attribution culturelle. in (2012).

50. Vostrovská, I., Kazdová, E., Kuča, M., Trampota, F. & Kolář, J. Kyjovice (okr. Znojmo) „Sutny“. LnK. MMK. Sídliště. Systematický výzkum. *Přehled výzkumů* **52**, 165–166 (2011).
